# Genetic variants in the p53 pathway influence implantation and pregnancy maintenance in IVF treatments using donor oocytes

**DOI:** 10.1007/s10815-021-02324-9

**Published:** 2021-10-07

**Authors:** Arturo R. Palomares, Adrián Alberto Castillo-Domínguez, Maximiliano Ruiz-Galdón, Kenny A. Rodriguez-Wallberg, Armando Reyes-Engel

**Affiliations:** 1 IVF Unit, Instituto de Fertilidad Clínica Rincón, 29730 Malaga, Spain; 2 Department of Biochemistry and Molecular Biology, Faculty of Medicine, University of Malaga, 29071 Malaga, Spain; 3 Laboratory of Translational Fertility Preservation, Department of Oncology and Pathology, BioClinicum J 5:30, New Karolinska Hospital, Karolinska Institutet, Visionsgatan 4, 17164 Solna, Stockholm Sweden; 4 Clinical Analysis Service, Virgen de La Victoria University Hospital, 29010 Malaga, Spain; 5 Karolinska University Hospital, Section of Reproductive Medicine, Novumhuset Plan 4, 14186 Stockholm, Sweden

**Keywords:** p53, Receptivity, SNP, MDM4, USP7, Implantation

## Abstract

**Purpose:**

Single-nucleotide polymorphisms (SNPs) in the p53 pathways have shown to play a role in endometrial receptivity and implantation in infertile women undergoing in vitro fertilization (IVF). The present study aimed to assess the influence of these gene variants over pregnancy success through a receptivity model in recipients of egg donation treatments, when factors such as age and quality of the oocytes are standardized.

**Methods:**

A nested case–control study was performed on 234 female patients undergoing their first fresh IVF treatment as recipients of donor oocytes. Genotyping of *TP53* Arg72Pro (rs1042522), *LIF* (rs929271), *MDM4* (rs1563828), and *USP7* (rs1529916) SNPs in the recipients allowed comparison of allele and genotype frequencies and their association with the IVF treatment outcome.

**Results:**

Grouped by genotypes, patients showed differences in IVF outcomes after the embryo transfer. Arg72Pro (rs1042522) gene variant was associated to changes in implantation and clinical pregnancy rates. The polymorphisms *USP7* (rs1529916) and *MDM4* (rs1563828) were associated to differential ongoing pregnancy rates and variable miscarriage events, respectively.

**Conclusions:**

This study highlights the association between gene polymorphisms related to P53 function and their influence over IVF reproductive outcomes. Arg72Pro variant may influence early events, as lower implantation rates were found in homozygous for Pro72 allele. By contrast, *MDM4* (rs1563828) and *USP7* (rs1529916) gene variants were associated with the later maintenance of pregnancy.

## Introduction

Pregnancy establishment requires important cellular and molecular changes through differentiation and proliferation processes [1]. Several biological processes supporting the embryo-maternal dialogue have been identified; however, the role of many interacting molecules and cells in the female reproductive tract during this critical process is still unknown, and it is not accessible for the clinical management in assisted reproduction technology (ART) treatments. Embryo implantation concerns a particularly exceptional mechanism between two cellular entities with different genetic backgrounds and also ontogenetic states, namely the trophoblast of the blastocyst and the epithelial endometrial cells of the uterine cavity, which interact synergistically for a common physiological purpose. The current research on the role of the endometrium in this synergy has focused mainly on its receptive potential. That research has given some insights about transcriptomic and microbiological conditionants in the recipient that could impair the implantation of the embryo [2, 3]. Endometrial receptivity tests are intended to assess solely the endometrial functional state. Additional strategies approaching genetic variability, such as the investigation of mutations, could provide useful data to track relevant conditions limiting the intercommunication between the endometrium and the embryo, and these could be studied using non-invasive methods.

The P53 family comprises a wide number of hyperconnected transcription factors associated to cellular apoptosis, cell cycle arrest, senescence, and DNA repair. The control of these processes by P53 protein products characterizes their function as a tumor suppressor [4–7]. Due to the maintenance of genome stability by these processes, P53 is known as “the guardian of the genome” [8]. Previous findings have revealed the implication of P53 in embryo implantation mediated by leukemia inhibitory factor (*LIF*) [9]. It has been suggested that this role must be a more primitive function than the most well known as a tumor suppressor [1, 10]. The role of *TP53* has been highlighted in embryo implantation, as the processes involved require intense transformation of cells that are additionally exposed to a very changing environment. It is thus suggested that a protective role of p53 could be relevant for maintaining the cells’ genomic stability at the embryo-maternal interface [11].

The influence of the P53 family in reproduction has been proposed to be dependent on *LIF* [12], whose critical role during the embryo-maternal dialogue has been demonstrated [13–15]. *LIF* participates in many diverse biological processes in the uterus involving cell proliferation, differentiation, decidualization, immune response, and vasculature remodeling [16]. Current data suggest that fluctuations on *LIF* levels in the endometrium could impair the establishment and maintaining of the pregnancy and that elevated and maintained *LIF* levels may improve endometrial receptivity [16, 17].

Additional gene variants related to the P53 pathway have been associated to reproductive success [18–20], and previous studies have assessed their influence over outcome of in vitro fertilization (IVF) treatments, however with conflicting results [18–24]. The available studies have mainly included patients that used homologous oocytes in IVF treatments; thus, important confounders such as infertility factors related to oocyte biology and female age have made it difficult to disentangle the influence of the gene variants investigated on the treatment outcomes [10].

For this study, we used the model of pregnancy establishment in women undergoing treatments using donor oocytes, to achieve a receptivity model for the investigation of biological features regarding embryo implantation when the recipient does not share genetics with the embryo to be implanted and to minimize the effect of factors intrinsically attained to oocyte competence, such as age.

For this genetic association study, we settled a list of candidate single-nucleotide polymorphisms (SNPs) related to P53 pathway, and these were genotyped to explore the reproductive outcome of IVF treatments in women recipient of donor oocytes. The selected variants consisted of the most known functional variant rs1042522 (Arg72Pro) in *TP53*; the rs929271 of *LIF* gene, whose function is essential during embryo implantation and which also seems to regulate P53 [13, 25]; the variant rs1563828 on Murine double minute 4 (*MDM4*) whose protein product inhibits P53 activity [26]; and the SNP rs1529916 located in the ubiquitin-specific-processing protease 7 (*USP7*) gene that shows a splicing variability according to genotype [26], as its protein product has also shown to modify P53 levels [27]. All the genes containing such selected variants were also selected as they are expressed in the endometrium robustly and consistently [28].

## Material and methods

### Study design and patient inclusion

A genetic association study with a nested case–control design was performed to assess the effect of selected candidate gene variants related to P53 pathway on the outcome of oocyte donation treatments and pregnancy progress. Patients undergoing IVF with donated oocytes were recruited for the present study at a private IVF unit. Included women were European Caucasian aged between 18 and 45 years undergoing the first oocyte donation treatment as recipients and receiving a fresh embryo transfer (ET) of at least two good-quality cleavage-stage embryos on day 2 or day 3. Women were excluded if they presented with uterine factor infertility, abnormal endometrial thickness, suspected hydrosalpinx, body mass index (BMI) > 30 kg/m^2^, or if there was severe male factor infertility in the partner. The patients provided a signed consent form for the IVF treatment and also for the genetic study and provided a DNA sample obtained from buccal swab.

### Controlled ovarian hyperstimulation (COH) in oocyte donors, oocyte retrieval, insemination, and embryo culture

Oocyte donors were European Caucasian healthy women with good physical and mental health aged 18–35 years, having regular menstrual cycles and a body mass index between 18 and 29 kg/m^2^. Donors were screened for known hereditary disorders, chromosome abnormalities, and infectious diseases. Donors were not accepted if they previously presented an abnormal response to gonadotropin, polycystic ovary syndrome (PCOS), endometriosis, more than one previous miscarriage, and any other medical condition that could compromise the success of the treatment.

Human recombinant follicle-stimulating hormone (hrFSH) and gonadotropin-releasing hormone (GnRH) antagonists were used for COH in donors, and hrFSH units were adjusted between 150 and 300 IU depending on age, BMI, antral follicle count (AFC), and ovary response. Oocyte maturation was triggered by administering GnRH analogue after having reached at least three dominant follicles ≥ 17 mm. Oocyte retrieval was done 35 h after the GnRH analogue administration. Denudation of cumulus oocytes complexes was performed by mechanical disaggregation after a brief exposure of hyaluronidase (40 IU/mL) in HEPES. Mature metaphase II oocytes obtained were inseminated using sperm from the male partner through intracytoplasmic sperm injection (ICSI) 2 h after denudation. Embryo culture was performed in low oxygen atmosphere 5% (O_2_) and 6% (CO_2_) using sequential commercial culture medium cleavage and blastocyst (SAGE).

### Embryo transfer

Embryo transfer (ET) to the recipients was performed on day + 2 or day + 3. Recipients underwent endometrial preparation based on suppression of ovarian function and increasing dosage of estradiol valerate tablets. From the day of donor’s oocyte retrieval, the recipients received micronized progesterone (P) vaginal pessaries for luteal phase support. The ET was performed only after confirmation of an endometrium with typical trilaminar pattern with a thickness of at least 6 mm. Embryos were selected for ET when at least two good-quality embryos reached grade A or B following Spanish Society for Studies on Reproductive Biology (ASEBIR) [29].

### Clinical outcomes

Pregnancy was determined by serum or urine tests detecting positive levels of human choriogonadotropin hormone β-hCG in oocyte recipients after two consecutive measurements in day + 11 or + 12 after ET, depending on cleavage stage of transferred embryos day + 3 or + 2, respectively (β-hCG ( +)). Clinical pregnancy (CP) was determined by ultrasonographical evidence of a gestational sac 5–6 weeks after ET. The absence of an ultrasound-identifiable pregnancy and a decrease in the levels of β-hCG represented a biochemical pregnancy loss (BPL). An ongoing pregnancy (OP) was defined as the presence of at least one viable fetus after week 12 of gestation. Miscarriages (M) were considered to be those gestational losses that occurred between weeks 4 and 20, having previously identified a gestational sac [30]. The implantation rate (IR) was calculated as the ratio between the number of gestational sacs found by ultrasound scans and the number of embryos transferred. Ectopic pregnancy was defined as those ultrasound observations of a sac outside the uterus [31]. Those recipients with no evidence of pregnancy after the treatment were defined as the nonpregnant group.

### DNA preparation and genotyping

Genomic DNA was isolated from buccal swab collected from the recipients and using the QIAamp DNA Mini Kit (QIAGEN, Valencia, CA). Genotyping process was outsourced to the genetic and proteomic services of Science and Technology School in the University of Pais Vasco (Bizkaia). TaqMan® Open Array Genotyping System (Applied Biosystems, UK) was used to identify *TP53* (rs1042522), *LIF* (rs929271), *MDM4* (rs1563828), and *USP7* (rs1529916). Allelic assignment was done using TaqMan Genotyper® (Applied Biosystems, UK). Positive and negative controls were included in each reaction assay to ensure the quality and concordance of results to validate them. A minimal calling rate of 80% was used to assign genotype in the SNP. The list of gene variants included in the panel is summarized in Table 1.

**Table 1:** Detail and description of candidate gene variants related to p53 pathway and selected for the present study

Gene	ID SNP	Variation	Consequence	EUR MAF	Call rate *N* (%)
*TP53*	rs1042522	G > C Arg → Pro	Missense	G: 72% C: 28%	207 (88.46)
*LIF*	rs929271	T > G	3′-UTR variant	T: 66% G: 34%	224 (95.73)
*MDM4*	rs1563828	G > A	Intron variant	G: 68% A: 32%	223 (95.30)
*USP7*	rs1529916	G > A	Intron variant	G: 69% A: 31%	220 (94.02)

Percentage of genotypes and allele frequencies for European population (1000 Genomes)

### Statistical analysis

The study was designed as a nested case–control in which cohort groups are established according to the results obtained after the IVF treatment. This enables the possibility of analyzing the incidence of the genotypes according to the population stratified by treatment results and by pregnancy progress. Positive or favorable outcome was settled as control and negative outcome as a case.

To assess any possible population bias compared to the general population, allele and genotype frequencies published for the studied SNPs in 1000 Genomes (Phase 3) were used as reference [32]. Differences between allele and genotype frequencies of 1000 Genomes population and the recipients, object of the study, were calculated by using chi-squared test that was also used to assess compliance of Hardy–Weinberg equilibrium between genotype frequencies of every SNP on each group. Pooled by genotype, differences between age and number of embryos transferred were calculated by analysis of the variance (ANOVA). No multiple test adjustment was carried out as the selection of SNPs was based on previous association studies.

The clinical treatment outcomes IR, CP, BPL, M, and OP were used to assess the treatment progression and also their variation between recipients grouped by genotypes through Fisher’s exact and chi-squared tests. When a variation in the number of recipients was present on any group representing a clinical significance, a logistic regression model was used to compute the odds ratio with a 95% confidence interval, and the age of the recipient was used as covariate and treated as a potential confounder for adjustment. Groups of genotypes for comparison were established by considering the possible recessive, dominant, codominant, and over-dominant models of inheritance. To provide the fittest model of interaction between genotypes, and assuming a total absence of a known direct biological influence, Akaike’s (AIC) and Bayesian’s (BIC) information criteria were used to select the degree of association for every genotype with treatment outcomes [33]. Statistical significance was considered when *p* < 0.05 2-sided for all the tests.

## Results

A total of 234 Caucasian European recipients undergoing an IVF treatment with donated oocytes aged 40.48 (± 4.37) were included in the study (Table 2). Most women attempted oocyte donation treatment due to advanced reproductive age (57.0%), ovarian failure (23.3%), previous poor response to gonadotropins in IVF attempts (12.5%), endometriosis affecting the ovary (4.0%), and unspecified reasons (3.2%). Assessment of β-hCG in blood or urine test performed 14 days after oocyte insemination allowed dividing the cohort into two groups: a total of 150 recipient had a positive β-hCG ( +), and the remaining 84 had a negative result β-hCG ( −) (Table 2).

**Table 2:** IVF outcomes of patients recruited for the study

Result	No	% (95% CI)
Cycles ET	234	-
β-hCG ( +)	150	64.10 (57.77–69.98)
Implantation rate	199/521	38.20 (34.12–42.44)
Clinical pregnancy	142	60.68 (54.30–66.72)
Ongoing pregnancy	119	50.85 (44.49–57.19)
Biochemical pregnancy loss	7	4.67 (2.28–9.32)
Miscarriage	17	11.97 (7.61–18.34)
Ectopic pregnancy	5	3.33 (1.43–7.57)

Data consists of overall group including all women recruited for the study

*CI* confidence interval

Allelic and genotype frequency distribution for the studied gene variants did not vary between the group of patients and the reference population (Caucasian European 1000 Genomes, Phase 3 GRCh37, public data), and the only differences were observed for the *MDM4* gene variant (rs1563828) (Table 3). The two groups β-hCG ( +) and β-hCG ( −) were compared for every SNP to assess deviation of Hardy–Weinberg (HW) equilibrium, and a significant variation between genotype frequencies was observed for the *MDM4* variant in the non-pregnant recipients (*p* = 0.006) (Table 3).

**Table 3:** Allele and genotype frequencies comparison between total study group and reference population* and the two largest groups of the cohort β-hCG ( +) and β-hCG ( −) recipients. Hardy–Weinberg equilibrium assessment

Gene (variant ID)	Allele genotype	Patients *n* (freq.)	Reference population* n (freq.)	^d^*p* value	β-hCG ( +) *n* (freq.)	β-hCG ( −) *n* (freq.)	^e^*p* value
*TP53* (rs1042522)	G	303 (0.73)	719 (0.72)	0.512	208 (0.77)	95 (0.66)	^a^0.016
	C	111 (0.27)	287 (0.28)		62 (0.23)	49 (0.34)	
	G/G	114 (0.55)	253 (0.50)	0.310	80 (0.59)	34 (0.47)	^a^0.034
	C/G	75 (0.36)	213 (0.42)		48 (0.36)	27 (0.38)	
	C/C	18 (0.09)	37 (0.07)		7 (0.05)	11 (0.15)	
*LIF* (rs929271)	T	283 (0.63)	666 (0.66)	0.262	184 (0.63)	99 (0.63)	0.863
	G	165 (0.37)	340 (0.34)		106 (0.37)	59 (0.37)	
	G/G	27 (0.13)	69 (0.14)	0.401	22 (0.15)	5 (0.09)	0.325
	G/T	92 (0.46)	202 (0.40)		62 (0.43)	30 (0.52)	
	T/T	83 (0.41)	232 (0.46)		61 (0.42)	22 (0.39)	
*MDM4* (rs1563828)	G	259 (0.58)	683 (0.68)	^a^3 × 10^−4^	171 (0.59)	88 (0.57)	0.777
	A	187 (0.42)	323 (0.32)		121 (0.41)	66 (0.43)	
	G/G	78 (0.38)	235 (0.47)	^a^0.001	53 (0.36)	^c^25 (0.43)	0.198
	A/G	83 (0.41)	213 (0.42)		65 (0.45)	^c^18 (0.31)	
	A/A	43 (0.21)	55 (0.11)		28 (0.19)	^c^15 (0.26)	
*USP7* (rs1529916)	G	298 (0.65)	689 (0.69)	0.699	186 (0.65)	112 (0.73)	0.100
	A	142 (0.35)	313 (0.31)		100 (0.35)	42 (0.27)	
	G/G	92 (0.46)	237 (0.47)	0.914	60 (0.42)	32 (0.55)	^ab^0.032
	A/G	91 (0.45)	219 (0.44)		66 (0.46)	25 (0.43)	
	A/A	18 (0.09)	47 (0.09)		17 (0.12)	1 (0.02)	

Comparison of the genotype and allele frequencies of the total cohort selected for the nested case–control study with European population (*in the left*), and also between recipients with pregnancy test positive and negative. Hardy–Weinberg equilibrium compliance was assessed per group and variant (*in the right*). Results displayed represent the value of chi-squared test (Pearson) and freq. (frequency)

^*^Frequency population of the 1000 Genomes Phase 3 GRCh37 for the selected gene variants

^a^ *p* < 0.05

^b^Fisher’s exact test

^c^Not compliant with Hardy–Weinberg

^d^ Patients vs reference population

^e^BP vs non-BP

The distribution of allele variants for the allele Pro72 (rs1042522) (C) showed a significant 12% increase in recipients tested β-hCG ( −) (*p* = 0.016) compared to β-hCG ( +), which resulted in an increase of homozygous Pro72 (C/C) recipients in the non-pregnant recipients, compared with the β-hCG ( +) group (Table 3). A significant increase of the A/A homozygous genotype of the *USP7* (rs1529916) gene variant was observed in β-hCG ( +) patients (Table 3). All the remaining SNPs showed no variation in allele and genotype frequency distribution between these two groups of patients (Table 3).

IVF treatment outcomes are presented individually per genotype in (Table 4). A negative trend for homozygous Pro72 recipients was observed by decreased implantation and clinical pregnancy rates (Table 4). Homozygous Pro72 recipients presented almost halved IR (21.95%) compared with heterozygous Arg72/Pro72 (42.34%) and homozygous Arg72 (39.31%) (*p* = 0.047); in the same line, these recipients also presented significantly reduced CP (38.89%) compared with the Arg72/Pro72 heterozygous (58.67%) and the Arg72 homozygous (68.42%). Recipients grouped by genotypes of the *MDM4* (rs1563828) and *USP7* (rs1529916) presented differences in the miscarriage and also in OP rates, respectively (Table 4).

**Table 4:** IVF outcomes between recipients grouped by genotype

Gene (variant ID)	Genotype	Age (mean ± SD)	NºET (mean ± SD)	IR (%)	CP (%)	BPL (%)	M (%)	OP (%)
*TP53* (rs1042522)	G/G	40.89 ± 3.89	2.18 ± 0.47	105/248 (42.34)	78/114 (68.42)	2/80 (2.50)	8/78 (10.26)	66/114 (57.89)
	C/G	40.61 ± 4.61	2.31 ± 0.46	68/173 (39.31)	44/75 (58.67)	4/48 (8.33)	3/44 (6.82)	39/75 (52.00)
	C/C	38.28 ± 5.11	2.28 ± 0.57	9/41 (21.95)	7/18 (38.89)	0/7 (0.00)	1/7 (14.29)	6/18 (33.33)
	*p* value	0.057	0.165	^b^0.047	^b^0.040	^a^0.333	^a^0.590	0.142
*LIF* (rs929271)	G/G	40.53 ± 3.36	2.19 ± 0.40	30/70 (42.86)	20/32 (62.50)	2/22 (9.09)	1/20 (5.00)	19/32 (59.38)
	G/T	40.31 ± 4.69	2.19 ± 0.46	84/221 (38.01)	60/101 (59.41)	2/62 (3.23)	5/60 (8.33)	53/101 (52.48)
	T/T	41.15 ± 3.51	2.26 ± 0.49	81/206 (39.32)	58/91 (63.74)	2/61 (3.28)	9/58 (15.52)	45/91 (49.45)
	*p* value	0.348	0.513	0.767	0.823	^a^0.453	^a^0.395	0.625
*MDM4* (rs1563828)	G/G	40.34 ± 4.73	2.21 ± 0.50	68/188 (36.17)	51/85 (60.00)	2/53 (3.77)	10/51 (19.61)	38/85 (44.71)
	A/G	40.49 ± 4.11	2.22 ± 0.44	87/198 (43.94)	61/89 (68.54)	4/65 (6.15)	3/61 (4.92)	55/89 (61.80)
	A/A	40.96 ± 3.79	2.22 ± 0.43	39/109 (35.78)	27/49 (55.10)	1/28 (3.57)	4/27 (14.81)	23/49 (46.94)
	*p* value	0.719	0.787	0.209	0.253	^a^0.882	^ab^0.044	0.072
*USP7* (rs1529916)	G/G	40.31 ± 4.76	2.21 ± 0.45	79/223 (35.43)	57/101 (56.44)	3/60 (5.00)	9/57 (15.79)	43/101 (42.57)
	A/G	40.50 ± 3.96	2.25 ± 0.49	85/216 (39.35)	62/96 (64.58)	4/66 (6.06)	4/62 (6.45)	58/96 (60.42)
	A/A	40.87 ± 4.83	2.30 ± 0.47	26/53 (49.06)	17/23 (73.91)	0/17 (0.00)	4/17 (23.53)	12/23 (52.17)
	*p* value	0.852	2.18 ± 0.47	0.179	0.225	^a^0.873	^a^0.082	^b^0.043

IVF outcomes of recipients grouped by genotype. Age (in years) and number of embryo transferred are represented by mean and standard deviation (SD) per group. *NºET* number of embryos transferred, *IR* implantation rates, *CP* clinical pregnancy, *BPL* biochemical pregnancy loss, *M* miscarriage, *OP* ongoing pregnancy

*p* values were calculated by chi-squared or Fisher’s exact tests

^a^Fisher’s exact test

^b^*p* value < 0.05

^c^Analysis of the variance (ANOVA)

Logistic regression analysis showed that recipients that were homozygous for Pro72 had a higher probability of not achieving pregnancy, and according to AIC and BIC parameters, the recessive model for the Pro72 allele fitted with better score between the other evaluated models (Table 5). The A allele of *USP7* (rs1529916) was associated to increased OP under a dominant model (Table 5). The over-dominant model fitted better for *MDM4* (rs1563828), and heterozygous A/G women presented with fewer miscarriages (Table 5).

**Table 5:** Logistic regression analysis of Arg72Pro *TP53* (rs1042522) for clinical pregnancy, *USP7* (rs1529916) for ongoing pregnancy, and *MDM4* (rs1563828) for miscarriage

Variant	Inheritance model	Genotype	Clinical outcome		OR (95% CI)	*p*-value	AIC/BIC
			Clinically pregnant Freq. (%)	Non-pregnant Freq. (%)			
**TP53** (*rs1042522*)		G/G	78 (60.5%)	36 (46.1%)	1.00	0.032^a.b^	275.2/288.5
	Codominant	G/C	44 (34.1%)	31 (39.7%)	1.54 (0.84–2.83)		
		C/C	7 (5.4%)	11 (14.1%)	**3.70 (1.30–10.56)**		
	Dominant	G/G	78 (60.5%)	36 (46.1%)	1.00	0.04^a.b^	275.9/285.9
		G/C–C/C	51 (39.5%)	42 (53.9%)	**1.81 (1.02–3.21)**		
	Recessive	G/G-G/C	122 (94.6%)	67 (85.9%)	1.00	0.027^a.b^	275.2/285.2^c^
		C/C	7 (5.4%)	11 (14.1%)	**3.09 (1.12–8.51)**		
	Over-dominant	G/G-C/C	85 (65.9%)	47 (60.3%)	1.00	0.42	279.4/289.4
		G/C	44 (34.1%)	31 (39.7%)	1.27 (0.71–2.28)		
			Ongoing pregnancy Freq. (%)	Non-pregnant Freq. (%)			
**USP7** *(rs1529916)*		G/G	43 (38%)	58 (54.2%)	1.00	0.042^a.b^	306.3/319.9
	Codominant	A/G	58 (51.3%)	38 (35.5%)	**0.48 (0.27–0.86)**		
		A/A	12 (10.6%)	11 (10.3%)	0.67 (0.27–1.67)		
	Dominant	G/G	43 (38%)	58 (54.2%)	1.00	0.016^a.b^	304.8/315^c^
		A/G-A/A	70 (62%)	49 (45.8%)	**0.52 (0.30–0.89)**		
	Recessive	G/G-A/G	101 (89.4%)	96 (89.7%)	1.00	0.92	310.7/320.9
		A/A	12 (10.6%)	11 (10.3%)	0.96 (0.40–2.28)		
	Over-dominant	G/G-A/A	55 (48.7%)	69 (64.5%)	1.00	0.018^a.b^	305.1/315.2
		A/G	58 (51.3%)	38 (35.5%)	**0.52 (0.30–0.90)**		
			Non-miscarriage Freq. (%)	Miscarriage Freq. (%)			
**MDM4** *(rs1563828)*		G/G	38 (32.8%)	10 (58.8%)	1.00	0.038^a.b^	102.9/114.5
	Codominant	A/G	55 (47.4%)	3 (17.6%)	**0.20 (0.05–0.78)**		
		A/A	23 (19.8%)	4 (23.5%)	0.63 (0.17–2.25)		
	Dominant	G/G	38 (32.8%)	10 (58.8%)	1.00	0.035^a.b^	103/111.6
		A/G-A/A	78 (67.2%)	7 (41.2%)	**0.33 (0.11–0.93)**		
	Recessive	G/G-A/G	93 (80.2%)	13 (76.5%)	1.00	0.075	107.3/116
		A/A	23 (19.8%)	4 (23.5%)	1.22 (0.36–4.09)		
	Over-dominant	G/G-A/A	61 (52.6%)	14 (82.3%)	1.00	0.014^a.b^	101.4/110.1^c^
		A/G	55 (47.4%)	3 (17.6%)	**0.23 (0.06–0.86)**		

Different inheritance models were used to compare the possible associations between genotypes and clinical outcomes. AIC and BIC values were used to rank the fittest models which were selected by the lowest value of both

*OR* odds ratio (significant in bold) with their respective *CI* (confidence interval)

^a^*p*-value (chi-squared test) (adjusted by age)

^b^*p* < 0.05

^c^Lowest AIC/BIC

## Discussion

The present study used a model based on donated oocytes to investigate the association between p53-related gene variants and the success of IVF treatments in women recipients of donor eggs. Our results showed that *TP53* Arg72Pro (rs1042522) was associated to higher likelihood of achieving successful embryo implantation and pregnancy establishment. Our study suggests that certain clinical outcomes could be associated to *USP7* (rs1529916) and *MDM4* (rs1563828) gene polymorphisms, which might affect the following events for maintenance of an ongoing pregnancy.

The group of recipients that did not achieve pregnancy showed a significant increase in the frequency of the allele Pro72 (Table 3), and IR were also decreased by half in recipients homozygous for this allele, which was associated with significant lower CP rates compared to recipients carrying at least one Arg72 allele (Table 4). *USP7* gene variant (rs1529916) was associated with later pregnancy events, and recipients carrying at least one A allele had higher OP rates than homozygous G/G. However, it is very interesting that the homozygous genotype A/A, which represents the lowest frequency not only among the studied population but also in the wide European people (1000 Genomes) [32], significantly increased in the group of recipient β-hCG ( +) (Table 3) and also presented with a trend to higher IR compared to the genotypes A/G and G/G, although this comparison did not reach statistical significance (Table 4). These data suggest that the *USP7* variant might also be associated with earlier events during pregnancy establishment and not only with events in the later stages. A lower risk for miscarriage was observed for heterozygous of the *MDM4* gene variant. These results reinforce the idea about the potential utility of p53-related gene variants as predictive markers in IVF and more specifically as an indicator of receptivity potential.

In this study, we did not screen for abnormal chromosomal errors in the embryos, which is a limitation of the study; however, to minimize this potential impact, our study was designed using the model of egg donor treatment, where donors are young and healthy women. Our results are in agreement with previous genetic association studies reporting a negative impact of Pro72 homozygous genotype on IVF treatments outcomes [18–20, 22, 24]. It is striking that IR varied between patients grouped by genotypes following a very similar trend in our study, in comparison to what has been observed in these previous reports. Variation of IR has been almost halved not only in women homozygous for Pro72 allele that were young (< 35 years) [19], but also in slightly older patients (37.9 ± 3.3 years) that were recruited due to previous history of recurrent implantation failure [20].

The available literature on possible associations between p53 gene variants and IVF outcome have so far had little or null impact in the practice of reproductive medicine. A main reason may be dependent on the previous inconsistent results reported [21] or that some studies have found an association between the Pro72 allele and a better reproductive outcome of IVF treatments [23]. A possible explanation is that the effect of the Arg72Pro variant seems to be moderate, and it may have gone unnoticed in both these previous studies due to recruitment of patients with various causes of infertility and a wide age range, both factors that could have both impacted oocyte competence. The recruitment of heterogeneous populations could additionally have masked the effect of these gene variants in the previous studies [10].

Our study aimed at investigating the impact of these gene variants of p53 on IVF outcomes, however using a specific model with good expected oocyte competence from donors and reduced dependency of female age factors, where the model of implantation and pregnancy establishment would be less impacted by these bias. Studies on IVF treatments in oocyte recipients have been previously conducted, but little information has been provided regarding the inclusion criteria of recipients and the selection criteria and age limit of the oocyte donors [19]. The recipients of our study were aged 40.48 (± 4.37) years, and the IVF outcomes varied according to genotypes similarly to the previous studies using homologous oocytes [18–20]; in addition, donors were aged within 18–35 years, and the influence of oocyte competence as a possible bias is expected to be decreased; hence, we could hypothesize that Arg72Pro variant may be associated with endometrial receptivity independently of the oocyte competence.

The genes *MDM4* and *USP7* are highly expressed in the endometrium, and they are critical regulators affecting P53 activity [28, 34, 35]. Although the functional impact of both selected intronic SNPs is unclear, they were also associated to infertile population in previous reports [19]. The data on association between (rs1563828) *MDM4* variant and lack of pregnancies has to be interpreted with caution, as it could be due to induced bias, as it did not accomplish HW equilibrium in the recipients that did not achieve pregnancy.

Biological processes by which p53 could mediate embryo implantation remain unknown. LIF has been postulated as a mediator of the p53 pathway, and it has been shown to regulate embryo implantation in mammals [36–38]; however, its role in pregnancy maintenance is not clear [14]. A functional implication of Arg72Pro has been widely studied, and previous experiments showed that the apoptotic activity associated to p53 is less efficient when the allele Pro72 is present [39]. A limitation of this apoptotic function could be a potential path by which Pro72 allele could reduce the pregnancy success as this role is needed for dialogue between the implanting blastocyst and receptive endometrium [40, 41]. Quantification of *LIF* expression indicates that it is two times higher in Arg72 homozygous carriers compared to that in Pro72 homozygous, which could impact differently success of pregnancy establishment [9]. Our results fitted with these previous observations, and the recessive model of the allele Pro72 is supported by these biological data. The higher IR in recipients carrying Arg72, of about twice that of recipients homozygous for Pro72, supports also the data on *LIF* functional expression that has been estimated for these genotypes [19]. Our results thus support the hypothesis that homozygous Pro72 recipients could present diminished *LIF* levels, and this could impair cellular and molecular dialogue during early embryo implantation, however without clear influence on the later progress of the pregnancy. By contrast, *USP7* (rs1529916) and *MDM4* (rs1563828) gene variants may have an impact in later stages. At present, the functional implications of these intronic changes are unknown, but it could be hypothesized that perhaps they could be linked to any other functional variant or at least mediate, under a genomic regulatory mechanism that potentially modify p53 activity and/or levels, in a manner that may impair the pregnancy maintenance.

Our data add to the current literature and our observations merit further investigation of the influence of p53 on embryo implantation and evaluate its predictive value for the clinical management of patients undergoing IVF treatments. As proposed by previous authors, controlled therapies based on exogenous administration of *LIF* or modification of p53 levels and/or activity could be a potential alternative to explore new research or future clinical improvements [10].

The present study provides support of a clinical association between polymorphisms located at genes involved in the P53 pathway and the success of IVF in recipients of donor oocytes, which was used as a model of pregnancy establishment independent of the oocyte genetic origin and reducing the bias of poor oocyte competence. Further studies are needed to clarify the biological context of our findings and to extend the knowledge of polymorphism of genes of the P53 pathway in different populations and ethnicities.
